# Hough Transform-Based Angular Features for Learning-Free Handwritten Keyword Spotting

**DOI:** 10.3390/s21144648

**Published:** 2021-07-07

**Authors:** Subhranil Kundu, Samir Malakar, Zong Woo Geem, Yoon Young Moon, Pawan Kumar Singh, Ram Sarkar

**Affiliations:** 1 Department of Electronics and Communication Engineering, National Institute of Technology Durgapur, Durgapur 713209, India; kundusubhranil14@gmail.com; 2 Department of Computer Science, Asutosh College, Kolkata 700026, India; malakarsamir@gmail.com; 3 College of IT Convergence, Gachon University, 1342 Seongnam Daero, Seongnam 13120, Korea; nunnengi@gachon.ac.kr; 4 Department of Information Technology, Jadavpur University, Kolkata 700106, India; pksingh.it@jadavpuruniversity.in; 5 Department of Computer Science and Engineering, Jadavpur University, Kolkata 700032, India; ram.sarkar@jadavpuruniversity.in

**Keywords:** dynamic time warping, handwritten word, Hough transform, keyword spotting, query by example

## Abstract

Handwritten keyword spotting (KWS) is of great interest to the document image research community. In this work, we propose a learning-free keyword spotting method following query by example (QBE) setting for handwritten documents. It consists of four key processes: pre-processing, vertical zone division, feature extraction, and feature matching. The pre-processing step deals with the noise found in the word images, and the skewness of the handwritings caused by the varied writing styles of the individuals. Next, the vertical zone division splits the word image into several zones. The number of vertical zones is guided by the number of letters in the query word image. To obtain this information (i.e., number of letters in a query word image) during experimentation, we use the text encoding of the query word image. The user provides the information to the system. The feature extraction process involves the use of the Hough transform. The last step is feature matching, which first compares the features extracted from the word images and then generates a similarity score. The performance of this algorithm has been tested on three publicly available datasets: IAM, QUWI, and ICDAR KWS 2015. It is noticed that the proposed method outperforms state-of-the-art learning-free KWS methods considered here for comparison while evaluated on the present datasets. We also evaluate the performance of the present KWS model using state-of-the-art deep features and it is found that the features used in the present work perform better than the deep features extracted using InceptionV3, VGG19, and DenseNet121 models.

## 1. Introduction

Automatic understanding of textual contents from handwritten document images is of great interest to researchers in the document-processing domain. This is primarily due to the wide use of handwritten documents for communication from the ancient ages. Even in today’s technology-enabled society, many people prefer to communicate using traditional pen and paper. Hence, researchers are developing methods to convert the textual contents of handwritten document images into machine-encoded forms. This conversion not only helps in storing the information contained by the text in a compressed way, but also assists in easy retrieval from a pool of such documents. Such efforts of obtaining the underlying machine-readable text from handwritten documents have opened up a new research domain known as handwritten text recognition (HTR). Despite some notable success in HTR as found in the literature [1,2,3], many uncertain problems associated with HTR remain unresolved. These problems are mainly related to variations in writing styles among different individuals, as well as that within a single individual, due to changes in mood, age, time, environment, or situation, etc. In addition to these, the aging of documents adds noises to digitized images and the removal of these noises is a hindrance to the solution of the problem. These (unsolved) issues led to alternative solutions that could at least make the documents searchable in their image forms.

Therefore, researchers have come up with a keyword spotting (KWS) method to make digitized handwritten document images searchable depending on the user-chosen word. This active research area helps in the automatic indexing of handwritten documents in their image forms. For a given query word, a KWS system attempts to locate all instances of it [4,5]. A KWS system attempts to locate a document page image from a set of untagged document images, using a ranked list of retrieved word images or instances with high similarity scores, for a given keyword.

This initiative—to search/retrieve digitized documents using word level information—is easier than performing it with a character level understanding, due to the increased complexities related to character level segmentation [6,7] that are imposed on this system. In this context, it is noteworthy that, in reality, indexing handwritten document images is not an easy task due to extremely varied writing styles of individuals. It becomes more difficult as we consider indexing of documents with historical importance since the generic HTR systems fail to provide the desired outcome [8].

In literature, depending on the search space, a KWS technique follows either a segmentation-based or a segmentation-free approach. A technique that follows the former approach assumes that the document images have been already segmented into words or text lines through some page segmentation method [9]. The use of the page segmentation technique generates different inputs (i.e., text line or word) for the system, and accordingly, a segmentation-based KWS technique classified into two categories: (i) text line based technique, which locates a query word within pre-segmented text lines [10,11]; and (ii) word-based technique, which assumes the document image is already segmented into word images and, therefore, only focuses on matching the query word image with the target words [12,13,14]. On the other hand, methods that follow a segmentation-free approach do not require any prior page segmentation technique, i.e., these methods do not possess any knowledge about the document structure or any such similar templates [15,16]. The segmentation-based technique is a better option between these two approaches if we consider computational cost and compare the performances of these two categories of works in the literature. The segmentation-free techniques take a long time to locate the specific regions of the document that may contain the query word image [8,17]. Here, it should be noted that the techniques that extract words from document images might be erroneous sometimes and can add some computational overhead if a segmentation-based approach is adopted. However, the state-of-the-art word extraction methods [18,19,20] that perform the tasks with good efficiency on complex documents, while consuming less time, could be used to get rid of these issues. For a similar reason, text line-based techniques are computationally more expensive when compared to word-based methods. Considering the above discussion, it can be safely said that one can segment a document image into words for fast retrieval of regions in document images that may contain the query word [5,8].

**Figure 1:**
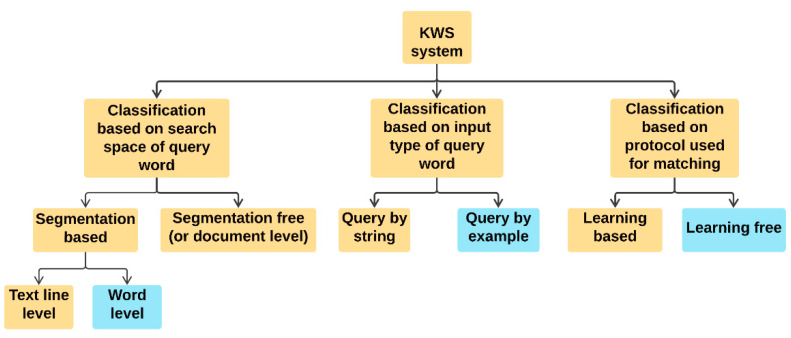
The taxonomy of the handwritten KWS system. The present method falls under the sub-categories shown in blue rectangular boxes.

Another taxonomy of KWS approaches is possible: query by example (QBE) [21,22,23] and query by string (QBS) [24,25]. This categorization is carried out depending on the input format of the query words provided to the system. In the QBE setting, the query word is an image, while in the QBS, set-up it is a text string. For matching of words, in literature, researchers have either followed learning-based [4,25] or learning-free [8,17] approaches. Techniques that include a training step are categorized as learning-based approaches, while in the learning-free scenarios, no such training step is involved. QBS approaches, in general, use a learning step to obtain the underlying character models, e.g., HMM models [2]. However, techniques that follow the QBE approach use the learning-free as well as learning-based approaches [4,8,26]. In this context, it should be noted that, although in the present work, we take the input query word as in text form as well as in image form, this technique follows a learning-free approach. The taxonomy of the KWS system described above is summarized in Figure 1. In this figure, the categories that we follow in this work are shown in sky-blue colored rectangular boxes.

A different set-up of experiments could be found in the works [4,26,27,28,29] where the use of multiple copies of image samples for each keyword could be found. Such set-ups are designed for a pre-defined set of keywords that need to be searched from a pool of words. In other words, the objective of these works is to decide whether a target word image belongs to the set of keywords (and if yes, then which one?). Multiple samples concerning each keyword used need to be prepared to create a trained module in a holistic way [4,26,27], or for tuning the parameters of the matching technique [28,29]. However, experimental set-ups mentioned in these works might not be possible in practical scenarios since such a system needs to be trained again if one wants to include a new keyword in the existing keyword set. Even, if the model is retrained, then one needs to collect multiple samples. Here, in our experiment, we consider a word image as a query word instead of considering a pre-fixed keyword. This restriction makes the KWS task more challenging since it is expected that information extracted from a single instance of a query word would represent all possible variations and can eventually identify the instances present in the collection of target word images.

In this context, we would like to mention that experimental performances are better for learning-based methods over the learning-free methods while considering QBE-based KWS. It is so because the presence of a single query word image and having no prior knowledge about the target word images while performing the spotting in a learning-free way limit the performance of this category of methods. Despite the low performance, the learning-free approach still draws the significant interest of the document research community due to the methodological simplicity, language independence nature, and non-requirement of additional example sets. In general, research interest in a learning-free approach emphasizes representing word images through robust feature descriptors with the capacity to narrow down the variations within the word images of the same class, and accordingly design some scoring techniques that can match two feature vectors. Here, it is worth mentioning that distance and sequence matching-based techniques have been widely used in literature. Additionally, for performing KWS, in this work, we considered a document image that was already pre-segmented into word images, using some word extraction techniques. The presence of word extraction methods [18,30] that can extract the word images from a document image efficiently motivates researchers only to focus on the word matching technique.

Keeping the facts (discussed above) in mind, we designed a KWS technique that follows the QBE approach. For this, we first applied a fuzzy system-based image contrast normalization on the word images (i.e., target and query word images) and then performed middle-zone normalization to handle skew and slant of input word images. Next, we partitioned the word images (both target and query word images) into several vertical fragments, which were equal to the number of letters in the query word. The number of characters was counted from the text encoding input of the query word, which was provided by the user to the system. After this, we extracted Hough transform (HT)-based features from each of these vertical fragments and, thus, we could calculate fragment-wise feature descriptors. Finally, we estimated the similarity score or matching score between the feature descriptors of a query word image and a target word image, by employing dynamic time warping (DTW).

To summarize, the main contributions of the present work are highlighted as follows:

- Generation of low-cost angular feature descriptors using HT. The number of features is equal to the number of characters in a query word, obtained from the machine-encoded text of the query word, multiplied by the number of angular bins considered in HT. To the best of our knowledge, this feature has been used here for the first time to deal with the KWS problem, and the dimension of this feature is much lower than that of state-of-the-art features.
- Formation of fuzzy membership function-based image contrast enhancement technique. The contrast enhancement technique helps in skipping the use of the edge detection method before applying HT.
- Application of word image normalization (i.e., slant and skew correction) at a grey level while the general tendency is to apply the same on the binarized image.
- Use of a variable number of vertical fragments (controlled by the number of letters in query word), as opposed to a fixed number of such fragments used by state-of-the-art methods.

The rest of this article is organized as follows: Section 2 contains a brief description of some state-of-the-art KWS methods that follow word level QBE. The proposed KWS technique is described in Section 3, which consists of four major sub-sections viz. pre-processing, vertical zoning, feature extraction, and matching of feature descriptors. The experimental results and corresponding analysis by highlighting the usefulness of the proposed method on various datasets are carried out in Section 4; and finally, in Section 5, we conclude our paper, by mentioning possible future extensions of the current work.

## 2. Previous Work

In this section, we briefly summarize some state-of-the-art works that have shown notable results in KWS in the QBE scenario. In this context, we would like to mention that the performance of QBE-based KWS techniques depends largely on the features used because the subsequent feature matching techniques perform well for better features. We found from the literature that a KWS method following the learning-free approach uses either single type features or a set of features of different types. Methods that use fixed-length and single type features, in general, utilize a simple distance/similarity measure technique for feature matching. The other category of methods demands a more refined algorithm for feature matching.

### 2.1. Methods That Use Single Feature Types

Methods that fall under the first category obtain good experimental outcomes and typically use angular information as a feature descriptor [8,12,31]. For example, Retsinas et al. [12] use the projection of oriented gradient (POG) feature descriptors. In this work, the authors first extracted the feature descriptors from the entire word image and vertically segmented image fragments, and then used the Euclidean distance metric for estimating similarity. The efficient use of the HOG feature descriptor has been noticed in the work proposed by Almazan et al. [31]. In this work, the similarity score has been estimated using the confidence score from the Support Vector Machine (SVM) classifier. Recently, the use of local gradient and Gabor-based features is found in the work proposed by Tavoli and Keyvanpour [27] for KWS in document images written in Roman and Arabic scripts. They use particle swarm optimization that includes multilayer perceptron (MLP) as a learner.

The use of the bag of visual words (BoVW) as a feature descriptor is another popular approach in KWS. Mostly, this approach has been used by the researchers to obtain spatial information of the word images [8]. BoVW-based techniques, in general, use three steps, namely, important feature point detection, feature extraction, and codebook conversion. In word spotting works, researchers mostly use key point extraction methods for feature point detection [32], whereas during the feature extraction phase, they preferred scale-invariant feature transform (SIFT) features over others. Codebook conversion represents the similar appearances of image patches over the word images. Aldavert et al. [33] describe many such KWS works in their survey paper. Along with the survey, they proposed a technique that was comparable to state-of-the-art techniques of that time. In this method, they use HOG feature descriptors as the bag of word (BoW) features and Euclidean distance as a similarity measure. The top two performers of the ICDAR 2015 competition on handwritten segmentation-based KWS track (Track IA and IB) [34] also use the BoW-based approach. Rothacker et al. [34], the best performer of the competition, use SIFT descriptor and the Bray–Curtis distance-based similarity measure, whereas Rusinol et al. [34], the first runner-up method, rely on integral HOG features with Euclidean distance-based matching technique. Zagoris et al. [35] employ an outlier detection-based algorithm to extract key points for the SIFT feature. They use gradient-based features in the process of keypoint detection whereas the nearest neighbor search technique has been used for the spotting of the words in segmentation as well as segmentation free approach. Recently, Yalniz and Manmatha [36] also use a similar approach for segmentation-free KWS purposes. In their work, they depend on their previously developed SIFT-based local visual features [37], whereas for matching, they use visterm-letter bigram dependencies.

We have found some methods in literature where researchers prefer to use the pyramidal histogram of character (PHOC) representation of word images for the said purpose. PHOC is a binary embedding for a word’s transcription. Almazan et al. [13] introduce PHOC representation of word images for KWS purposes. In this work, the Fisher vector was used as a visual feature, and the SVM model was used to label the character-like shapes. This method is suitable in both QBE and QBS scenarios as it uses the transcription of all the word images. Inspired by this work [13], Sudholt and Fink [14] exploit the concept in deep convolutional neural networks (CNNs), which is known as PHOCNet. Recently, another deep learning-based KWS technique was proposed by Barakat et al. [38] where a Siamese Network was used.

### 2.2. Methods That Combine Several Feature Types

The second category of methods combines multiple feature types to solve the KWS problem using learning-based [4,26,39] as well as learning-free [17,22,28] approaches. These techniques extract high-dimensional features, which make these a time-taking approach. By the term, “several feature types”, we indicate that features used to form a final feature set belong to more than one category (e.g., texture, angular, statistical, structural, and topological). For example, in the work [4], Malakar et al. make use of a modified HOG feature descriptor and a set of topological features, whereas Aghbari and Brook [26] use several structural and statistical features, extracted from an entire word image or its connected parts, to perform KWS, following holistic word recognition paradigm. In both methods, the authors use multiple copies of sample images for a pre-selected set of keywords to train a Multilayer perceptron (MLP) and a confidence score is used to decide whether an unknown word belongs to the keyword set or not. A similar experimental setup is also found in the work proposed by Khayyat et al. [39] where gradient-based features are used. However, they use a hierarchical classification strategy that uses SVM and regularizes discriminant analysis.

In the case of learning-free methods, the authors of [17,22,28] use a sequence matching algorithm for KWS. To represent a word as a sequence, in these works, authors extract column-based features, i.e., features are extracted from each column of the word images. For example, statistical features, eight different features (e.g., distance first data pixels from and bottom, the sum of pixel intensities, transition count, centroid position, and transition at centroid), and multi-angular feature descriptors are used in the works proposed by Rath and Manmatha [22], Mondal et al. [17,28] and Saabni and Bronstein [40], respectively. DTW is used to perform sequence matching in [22,40], whereas a flexible sequence matching technique is adopted in [28]. In another work, Kovalchuk et al. [41] extract histogram of oriented gradients (HOG) and local binary pattern (LBP) feature descriptors from word images segmented into a fixed number of vertical fragments. In their works, authors use the Euclidean distance metric for determining the similarity score. However, work [17] provides a comparative analysis of different sequence matching techniques for KWS in historical documents.

Though our present technique follows a feature descriptor matching technique, we only use an angular feature descriptor, which is generated using Fourier transformation. To the best of our knowledge, this feature descriptor has never been used in the literature of KWS. Besides, its dimension is much lower than that of the state-of-the-art feature descriptors mentioned here. The features are only extracted from vertical fragments that are generated using the count of the number of characters present in a query word image and with some vertical column overlapping. We use DTW, a popularly used sequence matching technique, where DTW finds similarity scores among the features extracted from the vertical fragments only.

## 3. Present Work

**Figure 2:**
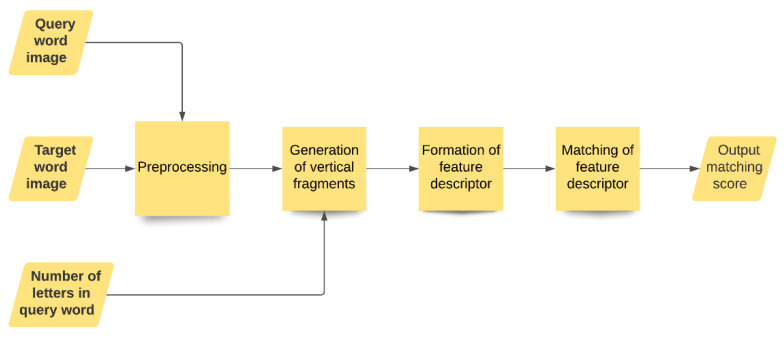
Block diagram showing the key modules of the proposed KWS method.

As mentioned earlier, in this work, we designed a recognition-free KWS technique where we followed the QBE setting. To be more specific, we arbitrarily chose a handwritten word image for a given query word, to conduct searching of the same from a pool of target words. Here, we first pre-processed the input word images (i.e., both query word sample and the collection of target word images), using a fuzzy membership function-based method. Next, the images were partitioned into several vertical fragments based on the number of letters present in a query word, which was then followed by the feature extraction and representation using a feature descriptor. Here, it should be noted that, during experiments, to obtain the number of letters present in a query word, we accepted text encoding of the query word from the user. In the end, we applied a DTW-based metric to estimate the similarity score or matching score between the feature descriptors of an example query word image and a target word image. We show the key modules pictorially in Figure 2, and all the modules are described in the succeeding subsections.

### 3.1. Pre-Processing

Many times, handwritten documents are prepared in a rush. Therefore, the words we write may be slanted and skewed. Moreover, due to aging, the quality of these documents get degraded. To deal with this, some pre-processing methods are performed in many handwritten document image-processing tasks. Here, we apply contrast normalization and word level skew and slant correction on grey level word images. These two preprocessing steps are motivated by the work by Retsinas et al. [8]. The pre-processing steps are divided into contrast normalization and middle zone normalization, which we describe in the following subsections.

#### 3.1.1. Contrast Normalization

In general, the input word image is considered a darker foreground (i.e., characters) on a lighter background. Hence, traditionally, binarization of the document images is considered the first step of pre-processing in the literature. However, binarization may be deceitful when each pixel’s intensity in the image is hardly differentiable as either foreground or background. Therefore, in this work, we did not directly convert an input image into its binarized form (as was done in the literature [4,8,29]); rather, we used the transformation of pixel intensities in an input word image by a soft assignment scheme that includes Sauvola’s binarization approach [42], first and later before extraction of HT-based feature extraction, we converted middle zone normalized images into their binarized forms using Otsu’s thresholding approach [43]. To determine each pixel’s intensity value in the transform domain, we used a fuzzy membership function. Let a contrast normalized image be denoted by $B_{cn}$. If the intensity value of a pixel at position (x,y) is outside the range [lb(x,y), ub(x,y)], it is directly assigned to either foreground or background pixel. We define lb(x,y) and ub(x,y) as in Equations (1) and (2):

(1) $$lb\left(x,y\right)=t\left(x,y\right)-\delta_{1}*\sigma \left(x,y\right)$$

(2) $$ub\left(x,y\right)=t\left(x,y\right)+\delta_{2}*\sigma \left(x,y\right)$$

**Figure 3:**
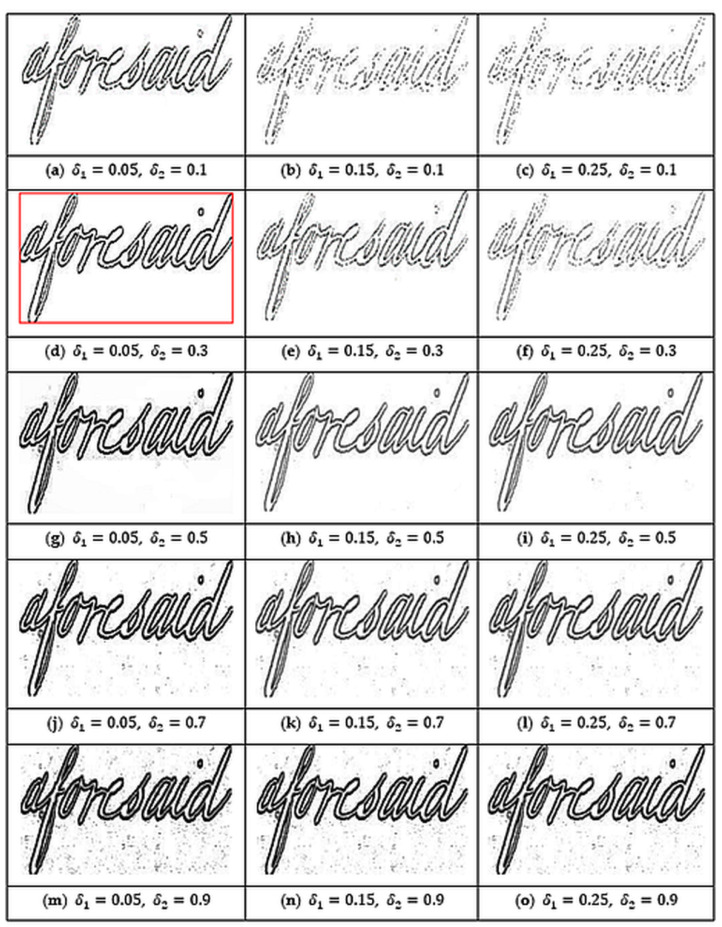
Effect of parameters $\delta_{1}$ and $\delta_{2}$ on the pre-processed image of the word ‘aforesaid’. It is seen that the output image (**b**) with $\delta_{1}=0.05$ and $\delta_{2}=0.3$ has the least background noise, coupled with the best prominence of the foreground against the background.

**Figure 4:**
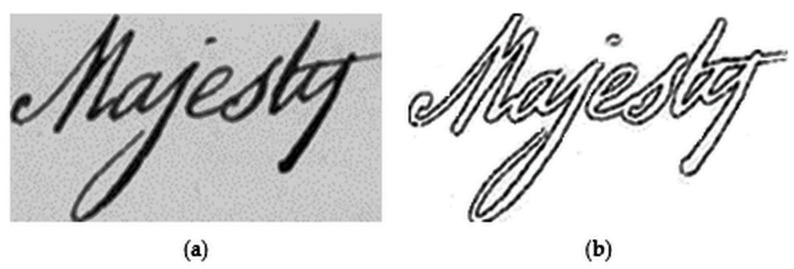
Effect of contrast normalization on an input handwritten word image. (**a**,**b**) represent input word image and contrast normalized word image respectively.

Equations (1) and (2), t(x,y) represent the Sauvola’s threshold value for the pixel at position (x,y), σ(x,y) is the standard deviation of the pixel intensities in the Sauvola window at position (x,y) and, $\delta_{1}\in \left[0,\;1\right]$ and $\delta_{2}\in \left[0,\;1\right]$ are two predefined constant values. In our work, we set the values of $\delta_{1}$ and $\delta_{2}$ experimentally as 0.05 and 0.3 respectively. Compared to the other sets of values in consideration, these values provide better results in pre-processing, as also depicted pictorially in Figure 3. Finally, the contrast-normalized image’s intensity value of each pixel (i.e., $B_{cn}\left(x,y\right)$) is given by Equation (3). Figure 4 shows the contrast-normalized output, corresponding to the input word image. It can be seen that the aforementioned process enhances the contrast between the foreground (characters) and the background, thereby, enabling us to obtain clear object (here, individual character) edges. Thus, the normalization process, in turn, makes it easier to extract angular features using HT later during feature extraction.

(3) $$B_{cn}\left(x,y\right)=\{\begin{array}{cc} 0, & 0\leq B\left(x,y\right)<lb\left(x,y\right)\;\\ 255\times \frac{B\left(x,y\right)-lb\left(x,y\right)}{ub\left(x,y\right)-lb\left(x,y\right)}, & lb\left(x,y\right)\leq B\left(x,y\right)\leq ub\left(x,y\right)\\ 255, & ub\left(x,y\right)<B\left(x,y\right)\leq 255 \end{array}$$

#### 3.1.2. Word Normalization

This step aims to generate normalized word images based on slant and skew angles estimated from the middle-zone of the word image. We performed this process on grey-scale word images. In this case, the input image is considered to have a linear upper and lower border, often called the upper line and the baseline, respectively. The middle zone estimation was attained by processing the horizontal projections of pixel intensities corresponding to the distinct angles, θ={−10°,−9°,…, 9°, 10°}. The horizontal projections were estimated using Radon Transform [44] due to its better skew angle detection capability [45]. Say L denotes the height of a word image and horizontal projection is denoted by $H_{\theta }\left(i\right)$, where i=1,.., L for a particular angle θ. We tried to find an upper line and a baseline (say m and n, respectively), such that $\sum_{i=m}^{n}H_{\theta }\left(i\right)$ was maximized while the middle zone height (n−m) was minimized. The problem at hand is therefore viewed as one, which finds the maximum contiguous sub-array, which may be solved in linear time using dynamic programming [46]. The angle θ, which gives the maximum value of the slope $S_{\theta }$, is the slope of the middle zone. $S_{\theta }$ is calculated using Equation (4), where a regularized parameter r controls the contribution of the two terms inside the summation.

(4) $$S_{\theta }=\underset{m,n}{\max}\;\left\{\frac{\sum_{i=m}^{n}H_{\theta }\left(i\right)}{\sum_{i=1}^{L}H_{\theta }\left(i\right)}-r\frac{n-m}{L}\right\}$$

There are two noteworthy aspects here, as described below:

(i) The length L is replaced by $L^{\prime }$, which accounts for about 95% of the total length of the horizontal projection [8]. If a and b, instead of m and n respectively represent the new boundaries that define $L^{\prime }$, we have the following relationship given by Equation (5).
  (5) $$L^{\prime }=b-a\;\mathrm{such}\;\mathrm{that}\;\frac{\sum_{i=a}^{b}H_{\theta }}{\sum_{i=1}^{L}H_{\theta }}\approx 0.95$$
(ii) The regularization parameter r is adaptive, in the sense that it can adjust its value depending upon the distribution of horizontal projection, thereby ensuring a narrower middle zone for words with elongated ascenders or descenders. The regularization parameter is formulated in Equation (6):
  (6) $$r=\frac{\sum_{i=a}^{b}H_{\theta }^{2}\left(i\right)}{{\left(\sum_{i=a}^{b}H_{\theta }\left(i\right)\right)}^{2}}$$

**Figure 5:**
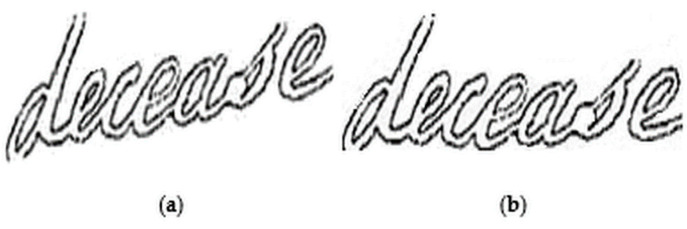
Effect of word normalization on contrast normalized word image. Here, (**a**,**b**) represent the actual word image and normalized word image, respectively.

After the middle zone is detected, the next step is to deskew the image. For this, we rotate the image at the angle $\theta =\underset{\theta }{\max}\left(S_{\theta }\right)$ using affine transformation [47]. Finally, we perform slant correction in the middle zone. For this, we use vertical projections of pixel intensities in place of horizontal projection in the process of slope angle detection. Images in Figure 5 show the effect of middle zone normalization.

### 3.2. Vertical Zoning

The word images (both search and target words), before feature extraction, are vertically segmented into several vertical fragments (say, $Z_{L}$) which is equal to the number of letters present in the query word image. The present system assumes that the value of $Z_{L}$ is provided to the system by the user. This vertical fragmentation or zoning helps in focusing on the features local to the specific regions of the query image. The purpose behind this is to match the query image with the target image, specifically concerning zones, thereby ensuring efficient comparison. Two aspects, to keep in mind, at this point are as follows.

1. An overlapping window of adequate size between two consecutive zones is included, to account for the variation in character size and handwriting style.
2. The number of columns in the search and target word images should be such that after zoning (considering the overlapping window), the number of columns in each zone is an integer. Therefore, a re-sizing of the images is required and based on $Z_{L}$, the number of columns is updated.

We assume that the number of columns in a pre-processed word image is C. Now, the number of columns (say, $C_{a}$) additionally included due to overlapping, is given in Equation (7), wherein, $C_{ol}$ indicates the number of columns being overlapped. Considering that $C_{a}$ number of columns are added; if there was no sharing of the overlapping columns between consecutive zones, the total number of columns without overlap (say, $C_{wo}$) would be according to Equation (8). Moreover, the number of columns required in the re-sized image (say, $C_{r}$) about $C_{a}$ is given by Equation (9), which is subject to the condition that $\underset{i}{\min}\left\{C_{wo}+i\right\}$ is perfectly divisible by $Z_{L}.$

(7) $$C_{a}=\left(Z_{L}-1\right)\times C_{ol}$$

(8) $$C_{wo}=C+C_{a}$$

(9) $$C_{r}=\underset{i}{min}\left\{C_{wo}+i\right\}-C_{a},where\;i\in \left[0,Z_{L}\right)$$

Now, the required number of columns for each zone of the re-sized image (say, $C_{z}$) is calculated using Equation (10).

(10) $$C_{z}=\frac{C_{r}}{Z_{L}}$$

Further, each zone can be defined in terms of its start column index (say, $j_{s}$) and end column index ($j_{e}$). For the $n^{th}$ zone $Z_{n}$, the related quantities, $j_{e}{|}_{n}$ and $j_{s}{|}_{n}$ (here, $n=1,\;2,\;\ldots ,\;Z_{L}$) are given by Equations (11) and (12) respectively.

(11) $$j_{e}{|}_{n}=nC_{z}-\left(n-1\right)C_{ol}$$

(12) $$j_{s}{|}_{n}=j_{e}{|}_{n}-\left(C_{z}-1\right)$$

**Figure 6:**
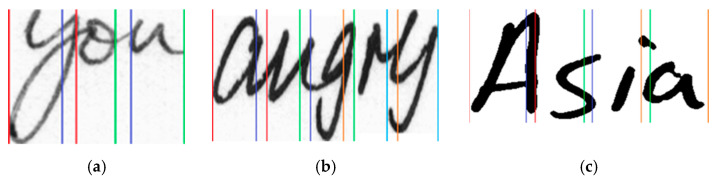
Pictorial representation of the vertical zoning process with overlapping columns considering three input images (**a**–**c**). Vertical lines of the same color indicate the beginning and end of a zone. Consecutive vertical zones always have an overlapping region in between them.

Vertical zoning, as mentioned here, is pictorially represented in Figure 6.

### 3.3. Feature Extraction

After the completion of pre-processing and zoning, in the next step, we extract orientation map-based feature descriptors from both the target and query word images. Here, we used HT to extract features from the word images; more specifically, the vertical segments of the word images, obtained by the process as described in Section 3.2. We should mention that many researchers have used HT to extract features for several image processing and pattern recognition tasks, such as finding strokes in geoscientific images [48], mammogram classification for early detection of breast cancer [49], face recognition [50], contextual line feature extraction for semantic line detection [51], detection of electric power bushings from infrared images [52], and many more. In general, in such applications, HT is used to find the straight lines in the image space. In the above-mentioned research works, the authors have looked for the top-most values in the Hough space (described later) globally. However, in the present work, we attempted to find the maximum number of data pixels associated with differently aligned straight lines, corresponding to each angular zone in the image space.

The HT uses the parametric equation of a line, defined by Equation (13), where the variables ρ and θ∈[−90°,90°) represent the perpendicular distance from the origin to the line and the inclination of the perpendicular line drawn from origin to the line to the positive x-axis.

(13) ρ=x cosθ+y sinθ

**Figure 7:**
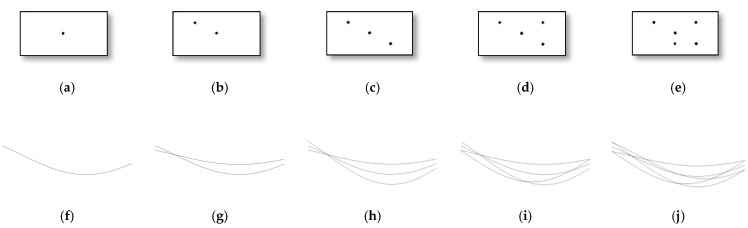
Pictorial representation of points in image space and Hough space. (**a**–**e**) show images containing 1–5 number of points respectively and (**f**–**j**) show corresponding lines in the Hough space.

The inputs to the HT are the image, the resolution ($\rho_{r}$) and the angle (θ) values, and the output is Hough space (H). In HT, a point in the image space is transformed to a line in the Hough space (see Figure 7a,f). If a straight line, perpendicularly inclined at angle θ to the positive direction of the *x*-axis, and at a distance ρ from the origin of the co-ordinate axes contains n data pixels in the image space, then in the Hough space n lines will pass through the point H(ρ, θ). The same is illustrated in Figure 7. From the figure, it is observed that if multiple points in the image space lie in a straight line, then the corresponding lines containing the pixels in the Hough space, intersect. $\rho_{r}$ and θ values determine the row count (say, r) and column count (say, c) of H along ρ and θ axes, respectively. The r value is estimated by Equations (14) and (15).

(14) $$r=2\frac{d}{\rho_{r}}+1$$

(15) $$\mathrm{where}\;d=\rho_{r}\times \lceil \frac{D}{\rho_{r}}\rceil \;\mathrm{and}\;D=\sqrt{{\left(n_{r}-1\right)}^{2}+{\left(n_{c}-1\right)}^{2}}$$

In Equation (15), the quantities $n_{r}$ and $n_{c}$ represent the counts of rows and columns in the word image or image zone, provided as input to the HT, while d represents the diagonal length. The expression ⌈·⌉ stands for the ceil function such that ⌈x⌉ maps x to the least integer equal to or greater than x. At this point, it is also noteworthy that the range of ρ is defined in terms of d as, ρ∈[−d,d].

The dimension of H is r×c. Initially, each $H_{ij}$ (i=1, 2, …, r and j=1, 2, …, c) is zero. Next, the value of ρ for each non-background point (determined during pre-processing as described in Section 3.1) in the image, is estimated, for every θ. The ρ value so obtained, is further rounded off to the nearest row number corresponding to ρ in the H. Next, the corresponding accumulator cell is incremented by$\;\rho_{r}$. After the values of ρ for all points in the image have been calculated, if $H_{ij}$ has a value of N, it indicates that N numbers of points in the ρ−θ plane lie on the line which is described by θ(j) and ρ(i). In our method, we use the default value for$\;\rho_{r}$ i.e., $\rho_{r}=1$ and θ∈{−90°,−75°, …, 89°} which means we have considered only 12 angular variations.

Once the H is obtained, a feature descriptor is generated, called a feature vector, F. F is a row vector of length c. Every $j^{th}$ column entry of F (i.e., $f_{j}$) is formulated in terms of H in Equation (16) as follows:

(16) $$f_{j}=max\left\{H_{ij}\right\}\;\forall \;i\in \left[1,r\right]$$

From Equation (16), and the illustrations given in Figure 7, it is clear that we extract the maximum number of data points about each angle that lies on straight lines. That is, if we were to visualize the Hough space in ρ−θ as a matrix, we would pick the highest-valued entry from each of the 12 columns (corresponding to each angle), thereby resulting in a vector that has length 12 (equal to the number of columns). This ensures that we take into account the maximum number of data points along each angular zone.

Moreover, the reason we have decided to go with a feature descriptor vector consisting of maximum entries from each column (i.e., each angle), rather than globally taking the top-most values of the Hough space, is that it allows us to span the entire range of θ from −90° to 90° uniformly. Thus, the feature vector would have complete information on the concentration of data points along differently aligned straight lines in all possible directions, rather than just the ones in which it is frequent or prominent, which would be the case if taken globally.

To extract the feature descriptor of an entire word, F is generated using Equation (16) for each vertical fragment of the word image and concatenated. For further detection, the feature descriptors corresponding to the query image, (say,$\;F_{Q}$) and to the target image (say,$\;F_{T}$) are compared, as explained in the following subsection.

### 3.4. Matching of Feature Descriptor Sequences

The final step is finding out to what extent a query word image and a target word image are similar. This is performed by matching of the generated feature descriptor. Multiple methods might be available for such matching. DTW, Hausdorff distance (HD), or discrete Fréchet distance (DFD) are some popular and frequently used techniques found in the literature. DTW, however, is much more appropriate for measuring similarity between two temporal sequences, varying in speed. In addition to that, it generates better results than the other alternatives mentioned above. The time complexity of the DTW algorithm is $O\left(L_{1}L_{2}\right)$ if $L_{1}$ and $L_{2}$ represent the lengths of the first and the second sequence, respectively. Assuming$\;L_{1}\geq L_{2}$, the time complexity of DTW can be written as $O\left(L_{1}^{2}\right)$.

DTW computes an optimal match between two input sequences, subject to the following conditions:

1. Every value from the first input sequence is to be matched with at least one value from the second sequence, and vice versa.
2. The value at the first index from the first sequence is to be matched with the value at the first index from the second sequence, although it need not be the only match.
3. The value at the last index from the first sequence is to be matched with the value at the last index from the second sequence, although it need not be the only match.
4. The mapping of indices corresponding to value from the first sequence to those of the second sequence must be monotonically increasing and vice versa.

The optimal match is the one, which satisfies all of the above conditions, while having the minimum cost, wherein cost is the sum of absolute difference values between each matched pair of indices. The matching score is obtained using Equation (17), where $f_{DTW}$ denotes the DTW function:

(17) $$score=f_{DTW}\left(F_{Q},F_{T}\right)$$

Lower the matching score, better the match between the query and the target word image.

## 4. Results and Discussion

The present work is aimed at segmentation-based, learning-free keyword searching through QBE. The experiments are performed on a machine with specifications as follows: an Intel Core i5-6200U at 2.30 GHz with 8 GB RAM.

### 4.1. Database Description

**Figure 8:**
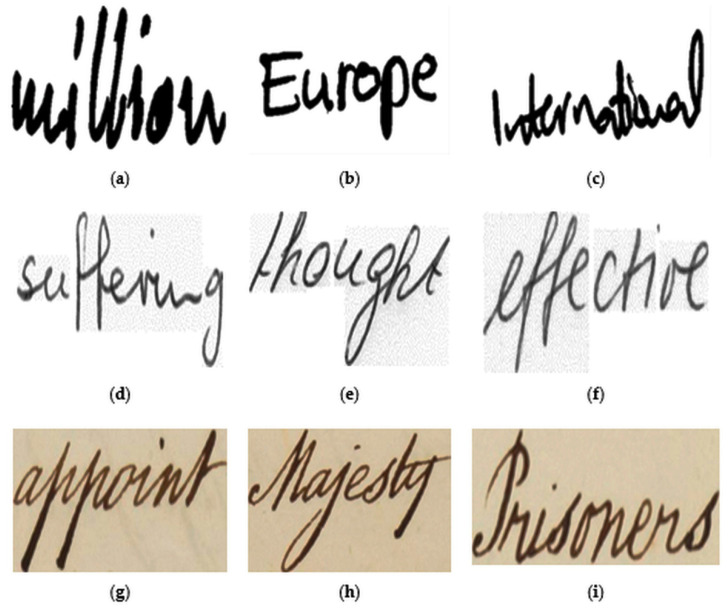
Samples of query images taken from (**a**–**c**) QUWI database, (**d**–**f**) IAM database, and (**g**–**i**) ICDAR2015 KWS database.

In the present work, we propose a KWS technique that takes the textual transcription of the query word from the user. For evaluation of the technique, we use three standard datasets, which are QUWI [53], IAM [54], and the validation set of ICDAR2015 competition on keyword spotting for handwritten documents (Track IA) [55], called the ICDAR2015 KWS database here. The QUWI database [53] is a large database of handwritten document page images. Part of the database is made public through ICDAR 2015 competition on multi-script writer identification and gender classification. This database contains 300 handwritten Arabic and 300 English document pages. In our work, we use English document page images only. We extract all the words from 25 documents (randomly chosen from 300 document pages) first, and then use these words as a target word set. The target word set contains 3449 word images, and as query words, we extract 75 word images from the rest of the document page images. The IAM database is an open-access large dataset of handwritten form-like document page images. The database contains segmented word images that are segmented using the page segmentation technique described by Zimmermann and Bunke [54]. Due to automatic segmentation, the segmented word images suffer from segmentation errors, such as under-segmented and over-segmented errors. Moreover, due to the extreme diversity of the writers, large variations are found in the writing samples. From the segmented word image database, we randomly select 9288 word images as the target word image dataset and 100 word images as the query word set. In this context, we should note that we have selected the query word images and target word images in a writer-independent way, and follow a database preparation strategy, such as ICDAR2015 competition on keyword spotting for handwritten documents (Track IA) [34,55]. The ICDAR2015 KWS database contains 3234 and 95 word images in the target and query word image dataset, respectively. Some examples of query words are shown in Figure 8.

### 4.2. Performance Measure

The performance is evaluated in terms of the mean average precision (MAP) score, which is popularly used and considered a standard evaluation technique in the literature, in regards to retrieval-based problems [13,56]. It is mostly used when the retrieved words are decided based on the ranking of the similarity score, such as the present one. It measures the strength of a retrieval system. MAP, for a set of queries, is the mean of the average precision scores for each query. To define the MAP score, we first define Precision (P) and Recall (R), as in Equations (18) and (19), respectively.

(18) $$P=\frac{\left|\left\{relevant\;words\right\}\cap \left\{retrieved\;words\right\}\right|}{\left|\left\{retrieved\;words\right\}\right|}$$

(19) $$R=\frac{\left|\left\{relevant\;words\right\}\cap \left\{retrieved\;words\right\}\right|}{\left|\left\{relevant\;words\right\}\right|}$$

Next, with the definitions of P and R, we define average precision, AveP as in Equation (20):

(20) $$AveP=\overset{n}{\underset{k=1}{\sum }}P\left(k\right)\Delta R\left(k\right)$$

where, k represents the ranks of correctly retrieved words within n number of retrieved words, while P(k) denotes the *p* value at certain cut-off (i.e., at kth retrieved words) in the list. ΔR(k) is the change in R from the items k−1 to k. Using the above definitions, now for the number of queries equal to Q, we can put forward MAP by Equation (21):

(21) $$MAP=\frac{\sum_{q=1}^{Q}AveP\left(q\right)}{Q}$$

### 4.3. Parameters Tuning

**Figure 9:**
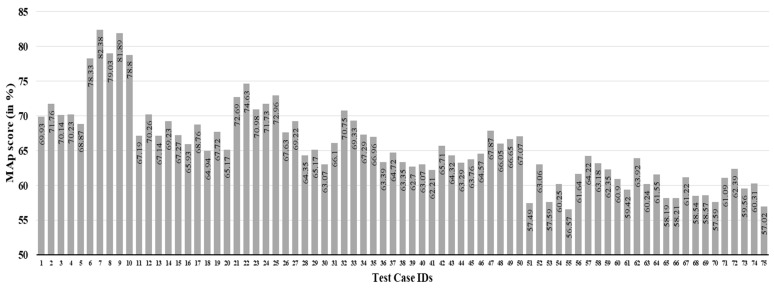
Comparison of the MAP scores for different values of $\delta_{1}$, $\delta_{2}$, and $C_{ol}$ on a small dataset, prepared for ablation study. The highest MAP score is obtained from test case ID 7, i.e., for the combination of $\delta_{1}=0.05$, $\delta_{2}=0.3$, and $C_{ol}=8$ (see Table 1). The test Case IDs in this figure are referred to as Test Case IDs in Table 1.

In this work, we used three parameters (Sauvola’s binarization constants (i.e., $\delta_{1}$ and $\delta_{2}$ and number of overlapping columns (i.e., $C_{ol}$) that need to be tuned. To set a proper value to these parameters, we perform an ablation study. We vary $\delta_{1}$ from 0.05 to 0.25 with a step size of 0.1, $\delta_{2}$ among 0.1, 0.3, 0.5, 0.7, and 0.9; while $C_{ol}$ from 6 to 14 with step size 2. Such choice of range for $\delta_{1}$ and $\delta_{2}$ is inspired by the fact shown in Figure 3. It shows that the further we increase $\delta_{1}$, the greater the presence of noise in the foreground, as well as background; while as we increase $\delta_{2}$, the contrast of the foreground against the background is reduced. Hence, the reason we limited our values of $\delta_{1}$ and $\delta_{2}$ to said ranges is that, beyond these points, although theoretically viable, pre-processing would result in either loss of foreground pixels or addition of much more noise in the image. This experimental setup gives rise to a total of 3×5×5=75 possible combinations. For each such set of $\delta_{1}$, $\delta_{2}$ and $C_{ol}$, we perform an ablation study on a small dataset containing 20 query word images and 1000 target word images prepared taking word images from the IAM dataset. We should note that this small dataset is completely different from the prepared dataset as described in Section 4.1. The average MAP score for the 20 query word images for each experiment is listed in Table 1. A pictorial representation of this performance is also shown in Figure 9. From this experiment, we can conclude that the combination of$\;\delta_{1}=0.05$, $\delta_{2}=0.3$ and$\;C_{ol}=8$, which is highlighted in the chart, gives the best MAP score. So, we use this set of parameters to conduct the rest of the experiments.

**Figure 10:**
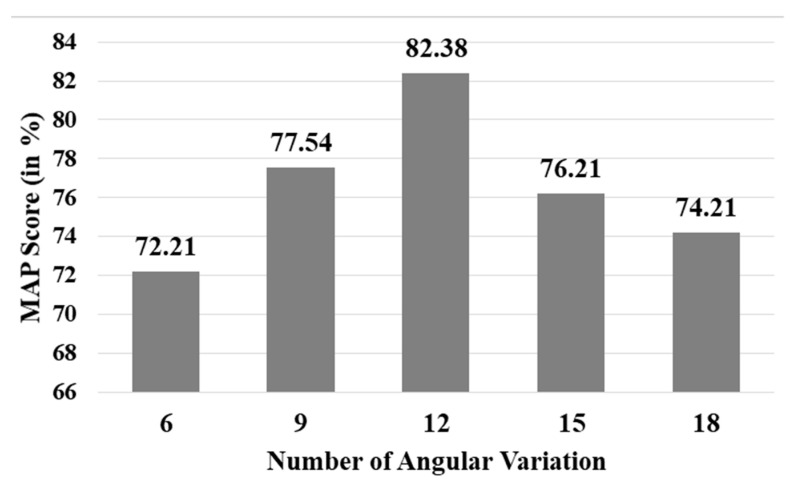
Comparison of the MAP scores with the varying number of angular variations in HT on the dataset, prepared for ablation study.

After fixing the above-mentioned parameter values, we performed another set of experiments to check the optimal resolution of HT space. In the previous experiments, we kept 12 angular variations from −90° to 90°, at an interval of 15° each. This time, we varied the number of angular variations. We considered five different variations: 6 (−90° to 90° at an interval of 30°), 9 (−90° to 90° at an interval of 20°), 12 (−90° to 90° at an interval of 15°), 15 (−90° to 90° at an interval of 12°), and 18 (−90° to 90° at an interval of 10°). The results are depicted in Figure 10. The results were generated on a small dataset (comprising 20 and 1000 query and target word images, respectively taken from the IAM dataset), specially prepared for parameter tuning as described above.

It is worth mentioning that we also tried experimenting by splitting the query word image into a fixed number of zones (4, 5, 6, and 7), just like reported in [8]; however, the results obtained thereby are not found promising. In the best case, we obtained a 75.29% MAP score using 6 word zoning on the dataset mentioned above. On the same dataset, our approach provided an 82.38% MAP score (see Figure 9 and Figure 10, and Table 1). Hence, a significant improvement in the result is observed on taking the number of letters in a query word image into consideration before its vertical zoning, in the manner explained in Section 3.2. This customization enables us to take into account the density of data pixels across the entire query image, so that it improves the accuracy further, when matching with target word images.

We further performed another set of experiments to see how the present preprocessing techniques worked (described in Section 3.1.1). For this, we first binarized the query and target word images directly using Otsu’s thresholding approach, and then applied out zone normalization. Next, we applied the Canny edge detection technique [57] to obtain the edge images, as shown in Figure 4. Such images are next passed to our feature extraction and matching protocol. The MAP score we obtained was 72.18% on the dataset, prepared for parameter tuning. The reason behind such lesser performance might be loss of data pixels or edge prominence nature during normalization and canny edge detection process and noisy images that might not always be handled by Otsu’s method during binarization. However, such loss of information does not occur during our process since we obtained edge images during the enhancement mechanism, and background noises are removed during that phase, and thereby use of Otsu’s binarization technique becomes effective.

### 4.4. Results

**Figure 11:**
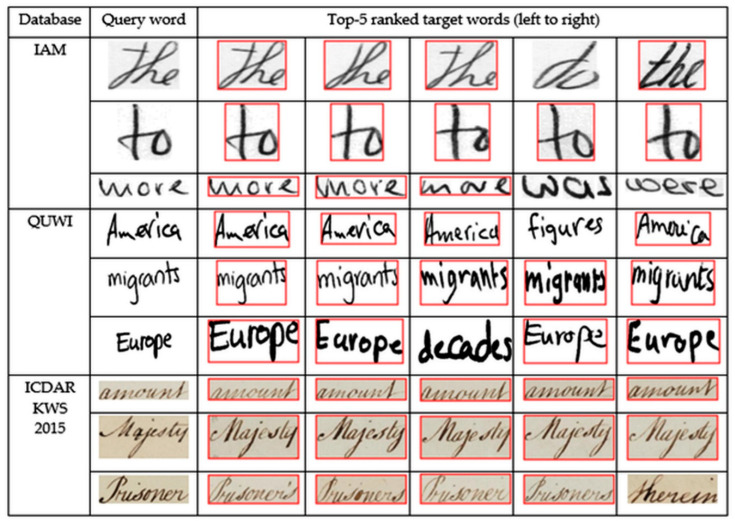
Top 5 ranked retrieved target words for the given query word taken from IAM, QUWI, and ICDAR KWS 2015 database. Words with red-colored bounding represent correctly retrieved word images while the rest represent wrongly retrieved word images.

We evaluated our method on the QUWI, IAM, and ICDAR2015 KWS databases and the experimental results are presented in Table 2. We obtained map scores 53.99, 86.40, and 45.01 on QUWI, IAM, and ICDAR2015 KWS databases, respectively. We also showed some examples of selected words by our method for some query words in Figure 11.

Moreover, we compared our method with the methods proposed by Mondal et al. [17], Mondal et al. [28], Malakar et al. [4], Retsinas et al. [8], and Majumder et al. [29]. For this, we implemented the methods proposed by Mondal et al. [17] and Mondal et al. [28] from scratch. For Mondal et al. [17], we used the classical DTW-based matching technique, while for Mondal et al. [28], we employed the flexible sequence matching (FSM) technique as proposed by the authors. We should note that we avoided the word image pruning from the target set, which was based on image dimension, used by the authors in these two works [17,28]. This was done in order to keep uniform performance calculation as well as to avoid any instance loss of query word image from the target word set during pruning. However, in the case of Malakar et al. [4], we first extracted the feature set used in this work and then applied DTW for finding the similarity score. For Retsinas et al. [8], we first extracted modified POG (mPOG) (POG process described in [12]) feature descriptor after segmenting the query and target word images into 5 and 6 vertical parts, respectively (configuration used by the authors), and then used single query matching scheme as this process is similar to ours. We used their functions for mPOG and the query -matching scheme, available at the GitHub link [58] in our setup. In the case of Majumder et al. [29], we used their code to test the performances of their model on the present datasets. Moreover, Majumder et al. [29] extracted deep features using pre-trained VGG16 [59] and HardNet-85 [60] deep learning models, pre-trained on the ImageNet dataset [61]. In addition to these two deep features, we also used three pre-trained models, namely, InceptionV3 [62], DenseNet121 [63], and VGG19 [59], which are trained on the ImageNet dataset [61], to extract deep features from the word images first, and then performed keyword spotting using DTW-based similar measure. All the methods were evaluated on the present datasets (refer to Section 4.1 for more details) and the results are recorded in Table 2. From these results, it is clear that the present method outperforms most of the learning-free KWS methods used here for comparison. Moreover, these results show that the use of angular features (i.e., presently designed features) in the current learning-free KWS is a better choice than that of deep features.

It is important to note the results shown in Table 2—that the length of our feature dimension is the least among the compared methods. The use of low dimensional features reduces the execution time, as the time complexity of DTW is $O\left(n^{2}\right)$, where n is the length of the feature dimension. This is because only the 12 angles from −90° to 90°, at an interval of 15° each, are considered to extract the feature values from HT. The maximum value in the Hough space, about each of the angles, is considered a feature value. These 12 features are extracted from several image patches, which are the same as the number of letters found in the query word, thereby enabling us to arrive at this low dimension of a feature descriptor. Hence, from this table, it is clear that the present method performs better than the methods we have compared with.

### 4.5. Performance Comparison on the Evaluation Set of ICDAR2015 Competition on KWS for Handwritten Documents (Track IA)

We further evaluated and compared the performance of the present KWS technique on the evaluation set of ICDAR2015 competition on KWS for handwritten documents (Track-1A) [34], much larger than ICDAR2015 KWS (validation set provided to the participants), used in the previous datasets. This test was conducted to access the performance of the present method on a relatively larger dataset. This dataset contained 1421 query word images and 15,419 target word images. The performances of the present method, along with the top two participating methods and the baseline method of the ICDAR2015 KWS competition [34], and the technique proposed by Retsinas et al. [8], are provided in Table 3. The results show that our model improves more than 15% MAP score than the best participating method in the ICDAR2015, while it performs close to the work proposed by Retsinas et al. [8]. Hence, it can be safely commented that the performance of the present method is comparable with the performances of these works.

### 4.6. Error Case Analysis

The target words that have been selected as top 5 choices are shown in Figure 9. It can be concluded from the results that our proposed algorithm relies completely upon the distribution of horizontal projections of angular bins, which is why it occasionally includes some false positives. There are quite a few examples to demonstrate the same. For instance, against the word “more”, the word “was” appears fourth among the top 5 retrieved words. The reason behind this is that the angular alignments of its constituent letters resemble that of the letters in the query word. The letter “w” as written, appears quite similar to the letter “m”, whereas the letter “a” is akin to the “o” and the “r” joined together. So is the case with “were”, which appears fifth in sequence. Once again, against “the”, it can be seen that the fourth match is the word “do”. This is explicable, considering that the letter “d” in “do” has been written in a very similar fashion to the “t” in “the”, thereby causing the similar distribution of horizontal projections of angular bins.

While it may seem that the false positives would only include target words, having the same number of letters as the query word, it is not necessarily true. The reason behind this is that the query word image is being divided into several zones based on its number of letters; and once done, the algorithm simply dissects each target word image into the same number of zones as that of the query word image. Thus, the target word image might still consist of two letters that are comparatively smaller, in the fashion in which they are written, thus making up for the area that one single letter in the query word image might have occupied. The reverse is also true. One of the letters in the “falsely” predicted match could be written in such spacious and enlarged forms that it might make up for the same room, as occupied by two adjacent letters in the query word image.

Among the three datasets used here for experimentation, it can be observed that the MAP score for IAM is quite high and stands out as compared to the values obtained for QUWI and ICDAR KWS 2015 datasets. A possible reason behind this could be the lack of variation among handwriting samples owing to a smaller number of writers in the dataset, due to which separate words do not stand out enough, thus leading to more false positives.

## 5. Conclusions

KWS is considered an important research topic among document image processing researchers. In the present work, we have proposed a learning-free KWS technique that searches the query word image from a pool of word images using the QBE approach. For this, we first applied some pre-processing methods on the images to get rid of noise components, and for middle zone normalization. After that, we divided the query word image and target word image based on the number of characters present in the query word. At this end, we accept the textual form of the query from the user to obtain the number of letters present in that word. Next, we extracted HT-based features from each of the segmented parts. Finally, we applied DTW to obtain a similarity score, thereby deciding the matching of words. To conduct the required experiments, we used three open-access databases: IAM, QUWI, and ICDAR KWS 2015. We obtained the MAP scores as 86.40%, 53.99%, and 45.01%, respectively, on IAM, QUWI, and ICDAR KWS 2015 databases. Our method performed better than some state-of-the-art learning-free KWS methods used here for comparison, in terms of MAP score, by using a much lesser number of features.

Although the present method performs well, there is some room for improvement. The results indicate that the present method does not perform well on the QUWI database, as compared to the IAM database. This might be due to a smaller number of writers in these databases, which leads to lesser angular variation within the writers. The inclusion of some structural and topological features might help in improving the performance of databases, such as ICDAR and QUWI, which have less variation. Moreover, the middle zone normalization technique sometimes fails to handle skewness in a word image properly. Therefore, the use of a better slant correction technique can be useful in improving the performance of the proposed method. Here, we used Otsu’s thresholding method to convert the normalized word images into their binarized form before HT-based angular feature extraction. However, this soft binarization technique might fail to perform well if the words are too noisy. Hence, in the future, a better binarization technique might be used to improve the overall performance of the present technique. A notable limitation of our work, as compared to the methods that use a fixed number of the segment, is that the number of vertical zoning is query word dependent, which, in turn, requires recalculation of features from target word images based on the number of letters in the query word. To overcome this issue, one can intelligently calculate and store the features for the target word set based on the possible letter–number variations that may occur in the query word set. Additionally, the number of query word samples used in this experiment is quite less. Therefore, experiments on more query word samples would help in establishing the robustness of the current technique.

## Figures and Tables

**Table 1:** Parameter tuning using ablation study to compare the MAP scores against various sets of $\delta_{1}$, $\delta_{2}$, and $C_{ol}$. The bold faced numbers represent the best score and values of the parameters- $\delta_{1}$, $\delta_{2}$, and $C_{ol}$ for which this score is obtained.

Test Case ID	$\delta_{1}$	$\delta_{2}$	$C_{ol}$	MAP	Test Case ID	$\delta_{1}$	$\delta_{2}$	$C_{ol}$	MAP	Test Case ID	$\delta_{1}$	$\delta_{2}$	$C_{ol}$	MAP
1	0.05	0.1	6	69.93	26	0.15	0.1	6	67.63	51	0.25	0.1	6	57.49
2	0.05	0.1	8	71.76	27	0.15	0.1	8	69.22	52	0.25	0.1	8	63.06
3	0.05	0.1	10	70.14	28	0.15	0.1	10	64.35	53	0.25	0.1	10	57.59
4	0.05	0.1	12	70.23	29	0.15	0.1	12	65.17	54	0.25	0.1	12	60.25
5	0.05	0.1	14	68.87	30	0.15	0.1	14	63.07	55	0.25	0.1	14	56.57
6	0.05	0.3	6	78.33	31	0.15	0.3	6	66.1	56	0.25	0.3	6	61.64
7	**0.05**	**0.3**	**8**	**82.38**	32	0.15	0.3	8	70.75	57	0.25	0.3	8	64.22
8	0.05	0.3	10	79.03	33	0.15	0.3	10	69.33	58	0.25	0.3	10	63.18
9	0.05	0.3	12	81.89	34	0.15	0.3	12	67.29	59	0.25	0.3	12	62.35
10	0.05	0.3	14	78.8	35	0.15	0.3	14	66.96	60	0.25	0.3	14	60.9
11	0.05	0.5	6	67.19	36	0.15	0.5	6	63.39	61	0.25	0.5	6	59.42
12	0.05	0.5	8	70.26	37	0.15	0.5	8	64.72	62	0.25	0.5	8	63.92
13	0.05	0.5	10	67.14	38	0.15	0.5	10	63.35	63	0.25	0.5	10	60.24
14	0.05	0.5	12	69.23	39	0.15	0.5	12	62.7	64	0.25	0.5	12	61.55
15	0.05	0.5	14	67.27	40	0.15	0.5	14	63.07	65	0.25	0.5	14	58.19
16	0.05	0.7	6	65.93	41	0.15	0.7	6	62.21	66	0.25	0.7	6	58.21
17	0.05	0.7	8	68.76	42	0.15	0.7	8	65.71	67	0.25	0.7	8	61.22
18	0.05	0.7	10	64.94	43	0.15	0.7	10	64.32	68	0.25	0.7	10	58.54
19	0.05	0.7	12	67.72	44	0.15	0.7	12	63.29	69	0.25	0.7	12	58.57
20	0.05	0.7	14	65.17	45	0.15	0.7	14	63.76	70	0.25	0.7	14	57.59
21	0.05	0.9	6	72.69	46	0.15	0.9	6	64.57	71	0.25	0.9	6	61.09
22	0.05	0.9	8	74.63	47	0.15	0.9	8	67.87	72	0.25	0.9	8	62.39
23	0.05	0.9	10	70.98	48	0.15	0.9	10	66.05	73	0.25	0.9	10	59.56
24	0.05	0.9	12	71.73	49	0.15	0.9	12	66.65	74	0.25	0.9	12	60.31
25	0.05	0.9	14	72.96	50	0.15	0.9	14	67.07	75	0.25	0.9	14	57.02

**Table 2:** Comparative results with state-of-the-art methods in terms of MAP score and feature dimension. The bold faced numbers represent the best scores while bold faced texts represent proposed method details.

Method	Feature Used	Length of Feature Dimension	MAP Score (in %)		
			IAM	QUWI	ICDAR KWS 2015
Mondal et al. [17], 2018	Column-based feature	8× image width	85.64	51.38	37.69
Mondal et al. [28], 2016	Column-based feature	8× image width	83.65	47.28	31.22
Malakar et al. [4], 2019	Modified HOG and Topological	186	81.50	52.12	35.27
Retsinas et al. [8], 2019	mPOG	2520 and 3024 for query and target word images respectively	75.21	52.73	**47.21**
Majumder et al. [29], 2021	Profile-based features	2× image width	82.10	50.43	32.19
Majumder et al. [29], 2021	Pre-trained VGG16 [59]	1024	80.35	42.18	17.12
Majumder et al. [29], 2021	Pre-trained HarDNet-85 [60]	2048	78.22	41.53	15.79
Szegedy et al. [62], 2015	Pre-trained InceptionV3	2048	44.98	30.18	12.59
Huang et al. [63], 2017	Pre-trained DenseNet121	2048	77.98	45.40	15.25
Simonyan and Zisserman [59], 2015	Pre-trained VGG19	1024	81.15	43.91	18.64
**Proposed method**	**HT-based angular feature**	**12× number of letters in the query word**	**86.40**	**53.99**	45.01

**Table 3:** Comparative results on the evaluation set of ICDAR2015 competition on KWS for handwritten documents (Track IA) with state-of-the-art methods in terms of the MAP score. The bold faced number represents the best score.

Method	MAP Score (in %)
Pattern Recognition Group (PRG) [34], 2015	42.44
Computer Vision Center (CVC) [34], 2015	30.00
Baseline Method [34], 2015	19.35
Retsinas et al. [8], 2019	**58.40**
Proposed method	56.12
